# Molecular Tools for Targeted Control of Nerve Cell Electrical Activity. Part II

**DOI:** 10.32607/actanaturae.11415

**Published:** 2021

**Authors:** D. V. Kolesov, E. L. Sokolinskaya, K. A. Lukyanov, A. M. Bogdanov

**Affiliations:** Shemyakin-Ovchinnikov Institute of Bioorganic Chemistry, Moscow, 117997 Russia

**Keywords:** optogenetics, chemogenetics, thermogenetics, action potential, membrane voltage, neurointerface, ion channels, channelrhodopsin, chemoreceptors, GPCR, neural activity stimulation, neural excitation, neural inhibition

## Abstract

In modern life sciences, the issue of a specific, exogenously directed manipulation of a cell’s biochemistry is a highly topical one. In the case of electrically excitable cells, the aim of the manipulation is to control the cells’ electrical activity, with the result being either excitation with subsequent generation of an action potential or inhibition and suppression of the excitatory currents. The techniques of electrical activity stimulation are of particular significance in tackling the most challenging basic problem: figuring out how the nervous system of higher multicellular organisms functions. At this juncture, when neuroscience is gradually abandoning the reductionist approach in favor of the direct investigation of complex neuronal systems, minimally invasive methods for brain tissue stimulation are becoming the basic element in the toolbox of those involved in the field. In this review, we describe three approaches that are based on the delivery of exogenous, genetically encoded molecules sensitive to external stimuli into the nervous tissue. These approaches include optogenetics (overviewed in Part I), as well as chemogenetics and thermogenetics (described here, in Part II), which is significantly different not only in the nature of the stimuli and structure of the appropriate effector proteins, but also in the details of experimental applications. The latter circumstance is an indication that these are rather complementary than competing techniques.

## INTRODUCTION

Minimally invasive methods of selective stimulation of the activity of nerve cells and brain structures hold a prominent place in the neuroscience toolkit. Part I of this review has focused on the most developed one, optogenetics, while Part II discusses the promising orthogonal approaches, thermogenetics and chemogenetics.

## THERMOGENETICS

Similarly to visible light, thermal energy propagates as electromagnetic oscillations and temperature is one of the key environmental factors interacting with biological organisms. The relatively narrow range of temperatures at which most cellular life-forms can function is determined by the thermodynamic and kinetic features of biochemical processes and facilitates the development of various evolutionary adaptations (such as thermotaxis, maintenance of a constant body temperature in homoiothermic animals, etc.) that are related to how temperature is perceived at the cellular and molecular levels [1, 2]. Thermoreceptors and other molecules that specifically capture temperature changes are typical of almost all living organisms [3]. This fact represents the foundation for the development of genetically engineered approaches to the manipulation of cell physiology and biochemistry using heating or cooling.

Thermogenetics is a relatively young group of methods where thermally sensitive, genetically encoded effector macromolecules are used to manipulate various physiological and biochemical processes in living cells. The thermogenetic approach can be viewed as an approach that is alternative or even orthogonal with respect to the one designated as optogenetic [4], but only with allowance for the fact that the former is significantly less commonly used. Thus, there currently exist less than a few hundred academic publications describing the application of thermogenetic methods.

An interesting difference between thermogenetics and optogenetics consists in the technological diversity of the methods used to activate effector molecules. The first method to appear, which remains the most commonly used, is heating of the entire model organism (this usually refers to heating insects in a special thermostat) [5, 6]. The second method to appear is the performance of local heating of tissues using magnetic nanoparticles that dissipate heat upon excitation by external fields. Thermal activati, on of the TRPV1 receptor by iron oxide nanoparticles induced by radio-wave irradiation is described in at least three studies [7, 8, 9]. The first of these publications demonstrated the principle underlying the method: Huang et al. [7] performed the excitation of cultured neurons expressing the TRPV1 receptor by radiofrequency radiation of ferrite nanoparticles placed on the cell surface. In the second study, Stanley et al. [8] successfully manipulated the blood plasma glucose level in mice with grafted tumors expressing the bioengineered insulin gene under the control of the Ca^2+^-sensitive promoter. The promoter was induced by calcium flux through the temperature-sensitive TRPV1 channel, whose molecule was labeled with nanoparticles using histidine tag antibodies [8]. In the third study, Chen et al. [9] stimulated neurons transiently expressing TRPV1 deep inside the brain tissue of living mice in a similar manner. More detailed information about the application of magnetic nanoparticles in thermogenetics has been provided in the topical review by Tay and Di Carlo [10]. Finally, the third method of thermogenetic stimulation involves infrared laser irradiation [11, 12, 13]. Bath et al. [12] developed an instrumental setup ensuring precise activation of Drosophila neurons and gave it an original name: FlyMAD (the fly mind-altering device). It is noteworthy that the nature of thermogenetic stimulation is responsible for both the fundamental limitations of the method and its potential advantages over optogenetics. On the one hand, the need to locally alter the temperature noticeably reduces the temporal resolution of the stimulation (this problem is partially solved using powerful IR lasers), while the approach involving overall heating of the object possesses such a drawback as virtual loss of spatial resolution. On the other hand, both infrared laser stimulation and radio-frequency excitation of nanoparticles are characterized by a high degree of stimulus penetration into the tissue (up to several millimeters), which makes thermogenetics noticeably advantageous over optogenetics in experiments aimed at studying such organs as the heart and the brain [4, 9, 10].

Although the thermogenetic approach is used relatively rarely as things stand, the repertoire of effector molecules and model systems associated with it is rather diverse and continues to grow. Thus, so-called RNA thermometers (RNATs) have been used as a tool for studying and modulating temperature-dependent gene expression in bacteria parasitizing homoiothermic animals [14]. The 3D structure of these wild-type sequences found in the 5’ untranslated regions of the mRNAs of some bacterial genes changes depending on the temperature. At low temperatures, the RNA thermometer inhibits mRNA translation by limiting the probability of ribosomal landing; contrariwise, translation is induced at higher temperatures. Another approach for the thermogenetic control of transcription is called IR-LEGO [15]. In this case, a living nematode C. elegans was exposed to IR laser irradiation to attain local activation of transgene (the GFP gene) transcription controlled by the heat-shock promoter hsp16-2. A similar irradiation scheme for the same model system has recently been used to demonstrate the FLIRT (fast local infrared thermogenetics) method [16]. In this case, the thermogenetic experiment was aimed at controlling protein activity, and the temperature-sensitive variants of myosin II, Delta and cyk-4 acted as targets.

Mutant GTPase dynamin, an expression product of the temperature-sensitive allele of the Drosophila shibire (shits1) gene, historically became the first thermogenetic effector in neurobiology [17]. Dynamin plays a crucial role in endocytosis regulation and, in particular, in synaptic vesicle recycling, while expression of its Shibire (G273D) variant inhibits vesicle activity due to depletion of the synaptic vesicular pool and blocking of synaptic transmission [18]. Reversible motor paralysis in animals in response to temperature elevation to 30°C was successfully demonstrated using targeted shits1 expression in Drosophila neurons [17]. Today, the shits1 allelic variant is a standard inhibitory effector in neurobiological studies focusing on Drosophila [19, 20, 21, 22, 23, 24].

Interestingly, chemoreceptors belonging to the IR and GR families are involved in thermoreception in insects [25, 26]. These molecules are ligand-specific, non-selective cation channels, while the molecular mechanisms that allow them to take part in the development of avoidance behavior in response to cooling or heating remain understudied. Nonetheless, one of the GR family receptors, Gr28bD, has become a progenitor of a fundamentally new class of thermogenetic actuators [26]. It has been found that thermostimulation of Xenopus oocytes and Drosophila motor neurons expressing Gr28bD results in the generation of a transmembrane cationic current that induces an action potential in neurons. Gr28bD was used as an activator of dopaminergic neurons when studying learning and memory in Drosophila [27]. Transient receptor potential channels (TRP channels) are the most important class of effector molecules used in modern thermogenetics, especially in relation to neurobiological problems [5, 28, 29].

**TRP channels**

**Fig. 1:**
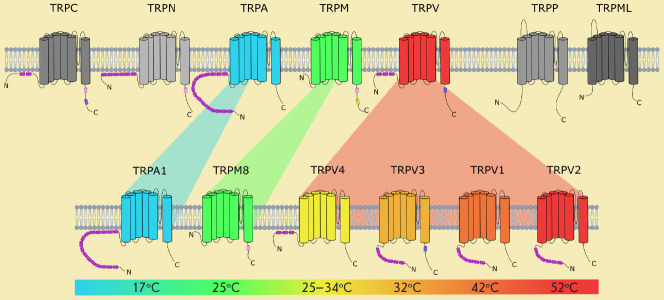
The TRP superfamily and temperature sensitivity of its chosen members. The top of the figure shows seven TRP-receptor families subdivided into two groups. In the bottom row, there are thermogenetically relevant molecules originating from three TRP families. The color scheme depicts the temperatures needed for the activation of the corresponding TRPs

TRP channels constitute a superfamily of ion channels residing on the plasma membrane of many types of animal cells. Approximately 30 types of TRP channels are currently known; they are clustered into seven families and share common structural properties (Fig. 1). All TRP channels consist of six transmembrane segments, show significant sequence homology within the family, and are characterized by nonselective cation permeation [30]. TRPs differ from other ion channels by an incredible diversity of cation selectivity and activation mechanisms. These proteins are involved in the functioning of all sensory systems (vision, gustation, olfaction, hearing, tactile perception, thermal sensitivity, and osmotic sensitivity). Hence, TRP channels mediate the cellular response to all the key classes of external stimuli, including light, sound, chemical substances, temperature, and mechanical force. Furthermore, TRP channels allow cells to sense changes in their immediate environment, such as changes in the osmolarity of a solution [30].

TRP channels are found in many multicellular organisms, including worms, insects, and vertebrates. According to the genetic organization and topology of their molecules, the entire superfamily of TRP channels can be divided into two large groups that include seven families (Fig. 1).

Nonselective permeation of cations (including Na^+^, Ca^2+^, and Mg^2+^) through the TRP channels becomes possible after activation. Ions entering nerve cells alter the membrane voltage and cause action potential generation. Interestingly, the conductance of TRP channels is three orders of magnitude higher than that of the channelrhodopsins involved in optogenetics [31].

TRP channels can be activated by various plant-derived substances, including those found in spices, e.g., in garlic (allicin), chili pepper (capsaicin), and wasabi (allyl isothiocyanate), as well as by menthol, camphor, peppermint, etc. TRP channels sensitive to temperature variation, or the so-called thermo-TRPs (Fig. 1), represent a highly relevant protein group to be used in thermogenetics. These channels are activated once a certain temperature threshold is attained. Thermo-TRPs are expressed in thermosensitive neurons and constitute the molecular basis for the organism’s response to thermal stimuli [30].

Four types of thermo-TRP channels activated by heating (TRPV1–4) and two thermo-TRP channels activated by cooling (TRPM8 and TRPA1, see Fig. 1) have been described. Upon heterologous expression (in HEK293 cells, CHO cells, and Xenopus oocytes), all six TRPs share the unique property of rendering cells temperature-sensitive. Each type of thermo-TRP channel has its unique temperature threshold of activation [30, 32]. The thermal sensitivity makes it possible for a neuron expressing thermo-TRP to be activated when the temperature is changed by 1–2°C [5, 33]. High ionic conductance makes these receptors particularly efficient neurobiological tools. Even at a lower expression level, thermo-TRPs cause a more stable depolarization compared to channelrhodopsins. The ability of thermo-TRPs to ensure reliable activation at moderate expression levels means that relatively "weak" promoters can be used in genetic vectors. Furthermore, low expression levels minimize the potential toxicity associated with the expression of exogenous proteins. Two tools based on thermo-TRP, rat TRPM8 (rTRPM8) [34], and the endogenous Drosophila receptor TRPA1 (dTRPA1) [5] (see details in the "Thermogenetics in Neurogiology" section) are currently used in Drosophila neurobiology. rTRPM8 is the "cold" channel activated at temperatures less than 25°C that is also sensitive to menthol [28, 35]. In routine experimentation, reliable activation of fly neurons using heterologously expressed rTRPM8 requires cooling down the animals to ≤ 18°C [34]. dTRPA1 is the Drosophila thermoreceptor that responds to heating and is involved in the induction of avoidance behavior at elevated temperatures in fly larvae [5]. Contrariwise, homologs of this receptor in mammals are sensitive to cold temperatures [36]. dTRPA1 is activated by moderate heating within a temperature range of 25–29°C or slightly higher [5, 36, 37, 38]. The temperature modes of rTRPM8 and dTRPA1 activation make these receptors poorly suitable for experiments with homoiothermic animals (and even their neuronal cultures). To date, most thermogenetic experiments with mammalian cells and tissues have been conducted using the "hot" vanilloid channel TRPV1 [7, 8, 9, 39], which is sensitive to capsaicin and is activated at appreciably high temperatures (> 42°C) [31, 40]. A few studies have reported on the use of other thermo-TRPs (TRPV2 and 3 in HEK293 cells [41], TRPV4 in a rat primary neuronal culture [11], and TRPA1 from the rattlesnake thermosensory apparatus in a murine primary neuronal culture [13]) as thermoeffectors.

**Thermogenetics in neurobiology**

While neurobiological optogenetics employs mice as the main model organism, neurobiological thermogenetics is almost exclusively the "territory" of Drosophila fruit fly [20, 42]. Over the past decade, researchers have achieved a real breakthrough in understanding how the nervous system of fruit fly functions by using a kit consisting of two thermo-TRP channels (rTRPM8 and dTRPA1 neuronal activators) and temperature-sensitive dynamin (Shibirets neuronal inhibitor). The thermogenetic approach was used for studying memory [21, 22, 23, 37], motor activity [19, 24, 34, 43], biological rhythms [38, 44], feeding [45, 46] and sexual [6, 47] behaviors, the connectome, and learning mechanisms in Drosophila [48]. Temperature-sensitive effectors were used in the original studies focused on the effect of microRNA expression [49] and the gut microbiome composition [50] on the behavior of fruit flies. A study kit for demonstrating 60 different types of thermogentically induced Drosophila behaviors has been designed based on the dTRPA1 thermoreceptor [51].

The application of the thermogenetic approach in vertebrate neurobiology has not been systematic thus far. In vivo activation of thermo-TRP in the neurons of the zebrafish Danio rerio [13, 52] and mice [8, 9] has been reported. As mentioned above, the principle of the thermogenetic activation method has been demonstrated for the culture of mammalian neurons in cellulo [7, 11, 13] and for acute slices of the mouse brain ex vivo [39].

**Limitations and perspectives of the method**

Modern thermogenetics is substantially inferior to optogenetics in terms of the spatial and temporal resolution of stimulation. Thus, thermo-TRPs activate neurons during several seconds [5, 33], which is probably indicative of the kinetics of tissue heating and cooling. When planning an in vivo thermogenetic experiment, it is necessary to bring the temperature mode of effector activation in line with the temperature optimum of the experimental animal. Going beyond the temperature optimum may induce the activation of the animal’s endogenous thermoreceptors and sometimes even cause thermal shock. This is especially challenging when working with homoiothermic animals, since the difference between normal body temperature and the temperature at which tissue destruction begins can be as small as 6–7°C. Heating (or cooling) of tissue with a high spatial resolution poses a much greater challenge than irradiation with visible light. On the other hand, when it becomes necessary to manipulate deep-brain structures or the nervous system in general, the thermogenetic approach can be preferable to the optogenetic one (as has been confirmed by its successful application in insect neurobiology).

Further advances in thermogenetics have been largely associated with the discovery of new effector molecules that are characterized in particular by rapid activation/inactivation kinetics and/or function within a temperature range of 38–42°C (in other words, well-compatible with the physiology of homoiothermal animals). The possibility of using thermogenetic neurostimulation for therapeutic purposes (e.g., for the functioning of cochlear implants) has been discussed in [53].

## CHEMOGENETICS

Chemogenetics is a family of methods involving the chemical stimulation of biological systems by small molecules mediated by actuators genetically incorporated into these systems. Chemogenetical actuators are characterized by (a) specific sensitivity to ligands acting as stimuli and (b) the ability to initiate physiologically/biochemically significant activity in response to ligand binding. Among the three approaches discussed in this review, chemogenetics is the one witnessing the most rapid development today. Thus, while in 2013 only about twenty studies employing chemogenetic tools (with few studies focusing on neurobiology) were published, at least 300 chemogenetic publications appeared in 2019 (they mainly involved in vivo experiments focusing on neurobiology). An explosion in interest towards tools for specific chemical stimulation started to register approximately in 2014–2015 and seems poised to increase in the near future. This boom in chemogenetics, partially caused by overall neuroscience "mobilization" (happening due to the advances in optogenetics, among other factors), is also substantially related to the enormous diversity of the mechanisms of small molecule stimulation.

The term "chemogenetics" per se can be interpreted widely. Below, we list the main chemogenetic approaches; from the ones less significant for neurobiology to the more significant ones.

Broch and Gautier [54] classify proteins/RNA fluorogens and small molecules acting as exogenous chromophores for these macromolecules as chemogenetic tools. Here, the dye-in-box principle of fluorescent labeling is implemented, when a non-fluorescent dye molecule that is capable of penetrating the cell binds noncovalently and highly specifically to a macromolecule genetically incorporated into the cell, thus acquiring fluorescent properties [55, 56]. A vivid example of the implementation of this concept is the FAST (fluorescence-activating and absorption shifting tag) system, whose initial form is represented by a monomeric, genetically engineered variant of the apo-form of the photoactive yellow protein (PYP) from a halophilic proteobacterium, Halorhodospira halophila, which forms fluorescent complexes with 4-hydroxybenzylidene rhodamine derivatives [57]. Chemogenetic tools for multicolor labeling [58], including far-red fluorophores [59], have been developed as part of FAST. As fluorescent tags, fluorogenic pairs have a number of advantages over both single-component, genetically encoded dyes (GFP and similar) and small-molecule organic fluorophores. In particular, they are typically characterized by high photostability and photo-fatigue resistance, which are critical in the context of advanced microscopy methods [60, 61].

Some researchers consider that chemogenetic methods include the design of artificial enzymes (mostly metalloenzymes) and control of their activity by biotin-(strept)avidin targeting [62, 63, 64, 65]. The principle implies delivery of biotinylated organometallic catalysts to a molecule of streptavidin or its variants. Chemogenetic optimization of the catalytic activity of such hybrid molecules can be achieved by combining the library of biotinylated catalysts with the library of streptavidin mutants [65].

A similar but more biologically relevant principle has been implemented in chemogenetics (or even chemogenomics) as a tool used for screening small-molecule libraries [66, 67, 68, 69]. This method usually implies that the biological model system is subjected to an impact from the target compounds, selection being performed with respect to a functionally significant parameter (e.g., phenotypic manifestation of enzyme activity). It allows one to identify the most active substance within the chemical library and, vice versa, the protein (or genotype) variant most sensitive to a selected individual substance. Yeast chemogenetic screening has made it possible to identify novel protein kinase inhibitors [66], histone acetyl transferase inhibitors [69], and fungicides [70]. The recent large-scale project [71] has characterized the resistome (i.e., a set of genes and their allelic variants associated with resistance to a certain substance) for the causative agent of malaria, relative to several dozen antimalarial drugs. Genetic determinants of multiple drug resistance have been identified.

The application of small molecules to control protein–protein interactions also conceptually refers to chemogenetic approaches. The chemically induced dimerization (CID) systems [72], which allow one to induce interaction between the target proteins fused to ligand-activated dimerization domains, are especially important here. CID systems based on homodimerization of the FKBP protein [73], heterodimerization of FKBP/FRB proteins [74], and their derivatives have shown good performance [75, 76]. These CID systems are used in neurobiology for reversible inactivation of synaptic transmission in vivo (in transgenic mice) by inhibiting the coalescence of synaptic vesicles [77]. The dihydrofolate reductase (DHFR) enzyme and its synthetic inhibitors (methotrexate and trimethoprim) are used in another family of chemically induced dimerization systems. For heterodimerizing targets, DHFR is combined with other ligand-binding proteins [78, 79]. Nanoantibodies (also known as nanobodies) based on this CID system, with their affinity to the target controlled chemically, are of significant interest [80, 81]. In particular, an antibody whose binding to GFP is switched on and off by NADPH and TMP ligands, respectively, has been reported [80]. This technology ensures chemically controlled reversible fluorescent labeling. Techniques for the computational design of protein molecules, which are expressed as two complementary fragments and can thus be associated upon ligand binding, are currently being developed [82].

The interaction between FKBP and its partner FRB, as well as the modulation of the activity of these proteins by small molecules (rapamycin, etc.), is applied not only in dimerization systems, but also in the chemogenetic regulation of the stability of the target proteins [83, 84, 85]. An interesting system for controlling the stability of the protein based on hepatitis C virus protease has been designed [86, 87]. When integrated into a chimeric protein, the viral polypeptide exhibits a default autoproteolytic activity, which is suppressed by the introduction of an inhibitor molecule into the system. Therefore, the chimeric protein retains its integrity and activity, as long as there is an inhibitor in the cell and it is degraded after the inhibitor is removed. Various pharmaceuticals have been successfully adapted to proteolysis inhibition, and this protein destabilization system has been shown to be promising in experiments involving transcriptional regulation, genome editing, and apoptosis.

Chemogenetic generators of small molecules come into general use. A vivid example is D-amino acid oxidase (DAAO), used to generate hydrogen peroxide in cells [88]. This yeast enzyme catalyzes the conversion of D-amino acids into the respective α-keto derivatives, accompanied by the release of a peroxide molecule [89]. Hence, almost any D-amino acid can be used to activate the H_2_O_2_ generator. DAAO is used as a chemogenetic effector in studies focusing on the activity of antioxidant systems [90] and cellular signaling [91] in cell cultures, as well as the effect of peroxide on cardiac activity in vivo [92]. In the aforelisted studies, DAAO was activated simultaneously with the monitoring of the peroxide level using fluorescent indicators.

Chemogenetic principles are used when designing fluorescent indicators of the membrane voltage. In some cases, voltage-sensitive dyes are targeted to the cell membrane using protein molecules (usually those binding covalently to these molecules) [93, 94] or even fluorogenically activated by membrane-bound enzymes [95]. In other cases, a plasma-membrane-anchored fluorescent protein acts as a FRET donor for organic fluorophore that migrates in the lipid bilayer in response to changes in the electrical potential [96]. Third, contrariwise, a microbial rhodopsin molecule acts as a voltage-sensitive unit, while its fluorescent signal is amplified due to resonance energy transfer from a bright fluorescent dye exogenously added to the cells [97, 98]. Such indicators are promising neurobiological tools; they are already being used today to monitor the electrical activity of neurons in vivo [98].

Chemical induction of gene transcription of bacterial enzymes is probably one of the first prototypes of chemogenetic methods [99, 100]. In turn, heterologous expression of bacterial enzymes acts as a basis for chemogenetic systems where pharmacologically relevant compounds modulate the activity of endogenous proteins in specific cell types. Thus, exposure of eukaryotic cells expressing bacterial β-galactosidase to daunomycin (daun02, a galactose derivative) was used as a model tool in tumor therapy [101]. The enzyme activity of β-galactosidase converts the pharmacologically inert daun02 into the daunorubicin antibiotic, which causes apoptosis. Experiments in the cells of a transgenic rat line where β-galactosidase is expressed under the control of the c-fos promoter are quite noteworthy in the context of neurosciences. Researchers employed the differential amplification of Fos (which is the endogenous transcriptional transactivator) expression in cocaine-susceptible neurons to selectively block calcium signaling in those cells. Therefore, infusion of daunomycin into the rat brain blocked ion channels (and, therefore, transmission of motor signals) only in cocaine-sensitized neurons [102]. Some natural neurotoxins show good potential for neurobiological application in chemically inducible expression systems. In particular, the tetanus toxin (TeNT) light chain inhibiting synaptic transmission by proteolytic cleavage of synaptic vesicle proteins [103], which is expressed in neurons under the control of tetracycline-sensitive regulatory elements, is used (together with tetracycline transactivator) as a reversible chemogenetic inhibitor [104, 105, 106].

Finally, there is a large group of chemogenetic effectors that is rather heterogeneous in terms of their structure and functions that is used almost exclusively in neurobiological research. We thoroughly characterized this group in the section below.

**Chemogenetic effectors for neurobiology**

All the effector molecules used in neurobiological chemogenetics can be subdivided into two types: ligand-gated ion channels and chemically activated G protein-coupled receptors [107]. The evolution of both types of molecular tools is most often achieved by using wild-type receptors towards the engineering of chimeric molecules optimized to address specific research issues.

Among wild-type ligand-gated cation channels, the TRP receptors already mentioned in the Thermogenetics section are used as chemogenetic effectors. We would like to remind the reader that these cationic channels are sensitive not only to temperature, but also to chemical agents. When establishing the role played by the TRPM2 endogenous receptor expressed in the mammalian hypothalamic cells in central control of body temperature, this protein was activated by a wild-type agonist, adenosine diphosphate ribose (ADPR), and its activity was modulated by a sensitizer, hydrogen peroxide [108]. Activation of the vanilloid receptor TRPV1 by capsaicin was used for neuronal excitation in the cell culture [109] and in the brain of transgenic mice in vivo [110, 111] (including studies on feeding behavior [112] and pain [113]). Menthol stimulation of neurons expressing the cold receptor TRPM8 was also described [109]. A substantial drawback of TRP channels as chemogenetic actuators consists in their presence in mammalian brain tissue as endogenous receptors, which can elicit a nonspecific response to stimulation. In that context, TRP knock-out mouse lines are used for in vivo studies [107].

**Fig. 2:**
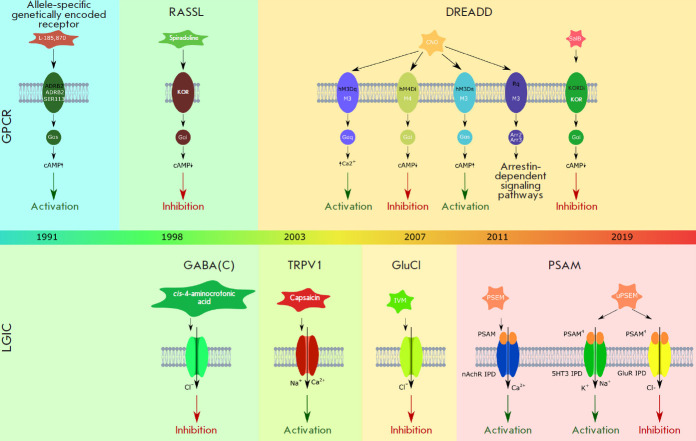
The timeline showing the emergence of diverse chemogenetic approaches. The main types of chemogenetic actuators, their wild-type predecessors (top panel: GPCR-based ones; bottom panel: the ones based on ligand-gated ion channels (LGICs)), and the molecular mechanisms providing their activation are shown

Cys-loop receptors constitute the most important family of chemically gated ion channels used in neurobiology [107, 114]. This family of pentameric molecules carrying a typical cysteine-rich structural unit that controls ion-pore permeability includes nicotine, glycine, serotonin, and GABA receptors, as well as glutamate-gated chloride channels [107]. Although wild-type Cys-loop receptors (in particular, GABA(C) and its agonist cis-4-aminocrotonic acid [115], as well as GluCl and ivermectin [116]), have also been used in single studies to control neuronal activity, their artificial variants, characterized by higher sensitivity [117, 118] and modified ligand specificity [119], as well as altered ionic selectivity [120], are used more commonly as neuromodulators. However, the family of PSAM chimeric module ion channels and their ligands (PSEM) is the most in-demand chemogenetic tool designed on the basis of Cys-loop receptors [107, 120, 121]. The first variant of a pharmacologically selective actuator module (PSAM) is the product of a genetic modification of the ligand-binding domain (LBD) of the α7 nicotinic acetyl choline receptor (nAchR), with the aim to reduce its affinity for acetylcholine and develop specificity to synthetic compounds that do not activate wild-type nAchR. These compounds are called pharmacologically selective effector modules (PSEMs) [122]. The features of Cys-loop receptors’ molecular organization (including structural independence of the ligand-binding domain (LBD) and the ion pore domain (IPD) [123]) have made it possible to perform the module engineering of PSAM-based receptors. Thus, the LBD selective to PSEM ligands was combined with the ion pore domains of other Cys-loop receptors [122]. In combination with the IPD of the serotonin 5HT3 receptor, the activated PSAM provides Na^+^/K+ fluxes into the cell, membrane depolarization, and neuronal excitation; in combination with the IPD of the nAchr receptor, it provides calcium flux into the cell; while in combination with the IPD of the glycine or GABA receptor, it ensures a Cl^-^influx accompanied by membrane hyperpolarization and silencing of neuronal activity (Fig. 2). Each of the chemogenetic modules (PSAM, IPD, and PSEM) can be subjected to further modifications with the aim to broaden the range of available ligands, increase specificity and ligand affinity, as well as ion pore conductance [107, 121]. The results of a large-scale study that focused on the rational design of a new PSAM4 activator specific to the anti-smoking drug varenicline, as well as a new family of uPSEM ligands characterized by subnanomodular affinity for PSAM4, were published in 2019 [124] (Fig. 2). The potential of these tools was demonstrated in in vivo experiments for the activation and inhibition of neuronal activity in the brains of mice and monkeys.

The PSAM/PSEM-based system of chemogenetic neuromodulation was used in a number of important studies that focused on the mechanisms of memory and learning [125, 126, 127, 128, 129], pain [68], the motivational effects of hunger and thirst [130], as well as motor and behavioral activity [131, 132] in vivo. The clinical and therapeutic potential of this approach was discussed in [133].

Identically to optogenetic tools based on microbial rhodopsins, ligand-gated ion channels (TRPs, Cys-loop) have an ionotropic mechanism of neuromodulation; i.e., they generate transmembrane ion currents that alter the polarization of the neuronal membrane. However, receptors with a metabotropic mechanism of activation (chemically activated G protein-coupled receptors, GPCRs) are used in chemogenetics much more widely than in optogenetics. It took approximately 20 years to create a pool of chemogenetic GPCRs suitable for in vivo neurobiological experiments, and several stages of their evolution can be distinguished (Fig. 2). The key amino acid positions associated with the specificity of adrenalin binding have been identified in the study of the molecular mechanisms of ligand recognition by β-adrenergic metabotropic receptors, and the resulting data were used to design receptor variants activated by synthetic catechol derivatives [134, 135]. That was how the first allele-specific, genetically encoded receptors appeared [121, 134]. Modified β-adrenergic receptors have not found application in neurobiology; however, altering ligand specificity by rational design has become a key concept in the engineering of the RASSL family of receptors that are activated solely by synthetic ligands [136, 137]. The first RASSLs were obtained by mutagenesis of the κ-opioid receptor (KOR) [136]. The modified KOR has lost sensitivity to endogenous peptide ligands but has become activatable by its synthetic agonist, spiradoline. The in vivo chemogenetic experiments with the early RASSL variants made it possible to modulate cardiac activity in mice [138]. Specific stimulation of gustatory neurons by RASSLs was later used to study the mechanism of sensation of the sweet, umami, and bitter tastes [139, 140]. The application of RASSLs in neurobiology is limited, mostly because of the sensitivity of endogenous opioid receptors to the RASSLs ligand spiradoline [107].

The drawbacks typical of RASSLs have been mostly eliminated in the next-generation chimeric GPCRs that became known as DREADDs (designer receptors exclusively activated by designer drugs, Fig. 2) [141]. These molecules are currently the most in-demand chemogenetic tools.

**DREADDs**

Systematic research into the molecular structure and activation mechanisms of wild-type GPCRs [142] has laid the conceptual foundation for designing chimeric molecules that can be activated by a pharmacologically inert substance [143]. The muscarinic acetylcholine receptor hM3 has been chosen as a pilot target for mutagenesis. After modification, hM3 has become highly selective to clozapine N-oxide (CNO) and has almost lost its affinity to the wild-type agonist acetylcholine [143]. This receptor has become the first member of the DREADD family and is known as hM3Dq, as it binds to Gq-type G proteins. CNO was chosen as a ligand, because this substance has favorable pharmacokinetics in both mice and humans; furthermore, it hardly activates endogenous GPCRs. The potential of hM3Dq was soon after demonstrated in in vivo experiments, where the receptor selectively activated mouse hippocampal neurons [144]. The mechanism of neuronal excitation caused by GPCR activation is much more complex than that for ionotropic receptors. Thus, hM3Dq activation induces phosphoinositol signaling, which enhances neuronal excitability, as well as the release of calcium cations from the intracellular depots, which in turn facilitates the driving of the Na^+^/Ca^2+^ antiport system depolarizing the membrane [107]. Like in the case of Cys-loop receptors, the "modular design" of GPCR molecules facilitates the design of new variants of the molecule. Thus, hM3Ds [145], a DREADD activating cAMP production in response to binding to CNO, was obtained on the basis of the aforementioned hM3Dq by replacing the intracellular G protein-binding module with the Gs-coupled module from the β-adrenergic receptor [146]. By analogy with hM3, other muscarinic receptors have been modified: so, the family of CNO-sensitive DREADDs has been extended. Along with hM3Dq/hM3Ds, it includes hM1Dq, hM2Di, hM4Di, and hM5Dq; some of them are now widely used in neurobiology [121, 143, 147]. In particular, hM4Di is an inhibitory effector that reduces cAMP production and ensures hyperpolarization of neuronal membranes mediated by potassium channel opening [143]. It was further demonstrated that hM4Di is also a potent inhibitor of synaptic transmission [148, 149]. Therefore, the DREADD family comprises both effectors activating neuronal activity and the ones inhibiting it, which influence the cell physiology via the three canonical G-protein signaling pathways (Gαs, Gαq, and Gαi). Furthermore, introducing an additional mutation to hM3Dq gave rise to DREADD[Rq(R165L)] that does not interact with G proteins but selectively triggers β-arrestin signaling [150]. The molecular design principles of DREADD have been used in the engineering of other GPCRs. Thus, modification of the κ-opioid receptor (KOR) made it possible to alter ligand specificity: instead of the wild-type agonist, psychoactive salvinorin A, its mutant variant KORDi functioning as a silencer of neuronal activity is activated by pharmacologically inert salvinorin B [151].

The strategies used to deliver the DREADD genes into target cells are generally similar to those used for delivering channelrhodopsins and other optogenetic effectors (see Part I of the review) and include transient expression using viral vectors and transgenesis [107]. DREADDs are activated solely by a target-specific chemical ligand (CNO), which is not found in the cells being stimulated and exhibits extremely weak activity against endogenous receptors. The advantages of using DREADDs for neurostimulation are as follows [121]:

(a) CNO can be delivered into an animal’s brain using both invasive techniques (injections) and via oral administration (with food or drinking water); no special technical facilities or manipulations (such as implantation of an optical fiber or the placing of an implant into the experimental animal’s brain) are required for DREADD activation;

(b) According to its pharmacokinetics, CNO exhibits sustained action on nerve cells (lasting from several minutes to several hours); so, experiments involving long-term stimulation can be performed. Furthermore, when being introduced into the animal’s organism, a DREADD ligand is appreciably uniformly distributed over tissues and reaches the deepest brain regions; so, the challenges related to the stimulation of large neuronal populations and difficultly accessible areas of the nervous tissue, which are typical of optogenetics, are ruled out in this case.

Therefore, while lacking a high spatial and temporal resolution and being barely suitable for the analysis of fast physiological processes, chemogenetic stimulation is an excellent tool for studying the effect of various chronic effects on cells or mimicking prolonged biological cycles (e.g., circadian rhythms).

Some difficulties related to the application of DREADDs are caused by the high doses of CNO required to attain sufficient stimulation intensity [152], the side effects associated with them [153], as well as a gap in our understanding of the molecular mechanisms of CNO penetration into the brain tissue. In 2017, it was demonstrated that DREADDs in rat brain are activated by clozapine formed metabolically rather than by CNO (which cannot easily penetrate the blood–brain barrier) [154]. These facts have driven researchers to design novel DREADD agonists characterized by better penetration characteristics into the brain and possessing a higher affinity for chimeric receptors [155, 156]. Advanced techniques for ligand delivery facilitating local penetration through the blood–brain barrier have also been proposed, in particular acoustic targeting [157] and chemomagnetic modulation using heat-dissipating nanoparticles [158].

**Neurobiological applications and the outlook for the method**

As we mentioned earlier, the past 4–5 years have witnessed a real boom in neurobiological chemogenetics. Most of the new studies employ DREADD-family receptors, in vivo experiments are performed, and the range of model systems used is as broad as that in optogenetics (from mice to monkeys). Interestingly, it took almost a decade for the approach related to designer chemoreceptors to become widespread in neurosciences. We attribute this to the complexity of the molecular mechanisms of neuronal stimulation using exogenous GPCRs. We will provide only some vivid examples of chemogenetic studies, as it is impossible to cover them all in a review.

The chemogenetic approach has allowed us to gain insight into the mechanisms of axonal regeneration [159], connectome organization and interaction between large neuronal populations [160, 161, 162], as well as to study the neurophysiological foundations of cognitive dysfunction on genetic models of schizophrenia [163, 164] and autism [165, 166]. In a number of studies, selective stimulation of neurons expressing DREADDs was used to investigate the behavioral effects of cocaine [167] and alcohol [168], as well as disruptions in the brain function in the offspring caused by alcohol consumption by a pregnant female [169]. Most of the "chemogenetic" publications have focused on deciphering the mechanisms of memory [170, 171, 172, 173, 174] and sleep [175, 176, 177, 178]. Some large studies have discussed the unusual associations between the functioning of the nervous and digestive systems: the role played by specific neuronal populations in the development of obesity [179], the gastroneural pathways of developing sweet taste preferences [180], and the effect of gut microbiota on the activity of sympathetic neurons [181]. In a recent elegant study, DREADD receptors helped to uncover an association between stress and the graying of hair [182]. The largest and most significant cluster of research projects employing the chemogenetic toolkit is related to the study of the neurophysiological determinants of animal behavior. These projects cover the traditional topics (such as feeding [183] and defensive [184] behavior and attention [185]), as well as specific behavioral patterns such as parental care [186] and mother–infant vocalization [187]. The chemogenetic tools have found application even in the study of the mechanisms of cat odor perception by mice [188, 189].

The question related to the clinical and therapeutic use of chemogenetics is being pondered. This refers to both the GPCR-based receptor–ligand systems [178, 190] and ligand-gated ion channels [133].

The incredible diversity of wild-type chemoreceptors (both their chemical specificity and activation mechanisms) opens up broad prospects for further development of the chemogenetic approach.

## COMBINATIONS OF THE APPROACHES

Thus, we have discussed the three modern approaches to controlling biochemical processes (while placing emphasis on control over the activity of nerve cells), as well as the molecular tools related to the implementation of these approaches. Each of them (the optogenetic, thermogenetic, and chemogenetic approaches) has its own merits and flaws, and the merits of one approach are often complementary to the flaws of another. This allows one to use a more efficient and relevant tool in each specific case and even combine different principles of cell manipulation in a single model system. Here, a role is played by the orthogonality of stimulation mechanisms; (e.g., the short-term optical stimulation through ion channels and prolonged chemical stimulation through G-protein signaling can obviously be mutually complementary.)

Indeed, some examples of complementary use of opto- and chemogenetics can be found in many neurobiological experiments. Thus, simultaneous in vivo optogenetic and chemogenetic stimulation is used to study the mechanisms of motivation [191] and behavioral adaptations [192, 193], to identify the role played by the sodium cation in circadian rhythm regulation [194] and the role of microglia in the regulation of myelination [195], as well as to study epilepsy [196], sleep physiology [197], regulation of feeding behavior [198], and pain perception [199].

Meanwhile, there are systems where the principles of optical and chemical stimulation are intertwined at the molecular level to give rise to actual hybrid molecular tools, rather than individual ones.

The first example of this kind might date back to the early days of neurobiological optogenetics, when hippocampal neurons expressing the ligand-gatedion channels TRPV1 and P2X2 were successfully stimulated by photoreleaseable ligands (capsaicin and ATP, respectively) in 2003 [109]. Later, hybrid photochemical stimulation of the P2X2 channel was used in vivo to control Drosophila behavior [200]. Several systems of reversible optochemical stimulation based on covalent protein modification by a small molecule linked by a photoisomerizable (azobenzene) group have been described. In one case, photoisomerization made it possible to open (using long-wavelength light) and close (using short-wavelength light) potassium channels [201], while in the other case, it allowed reversible ligand presentation to the ionotropic glutamate receptor (iGluR) [202]. Further development of this approach involved a modification of endogenous potassium channels and photochemical stimulation of the rat neurons mediated by them in cellulo and ex vivo [203], as well as the emergence of new "designer" potassium channels [204], acetylcholine [205] and glutamate [206] receptors with the same principle of activation. The latter receptors (belonging to the LiGluR family) were used in in vivo experiments [206, 207]. In 2020, a photochemically activated GPCR, the endogenous metabotropic glutamate receptor (mGluR2) capable of reversibly stimulating neurons, was first reported [208].

The Mito-FAP fluorogen activating peptide delivering the MG-2I photosensitizer into mitochondria also belongs to chemo-optogenetic effectors [209].

Finally, the BL-OG (BioLuminescent OptoGenetics) system, where the effector is a fusion protein (luminopsin) consisting of luciferase and channelrhodopsin, is the most "elegant" implementation of the hybrid photochemical approach to the control of cell activity [210, 211, 212, 213, 214]. A rhodopsin molecule is activated by luciferase luminescence; in turn, its induction and emission intensity can be adjusted by composition and the amount of cofactors (luciferin and its transporter) added to the system. The BL-OG system can be metaphorically characterized as a system for brain-targeted delivery of light.

## CONCLUSIONS

Optogenetics is a mature and extremely efficient method that has been widely recognized throughout the academic community. Thus, in 2010, Nature Methods recognized optogenetics as the Method of the Year [215], and Science included the technique into its "Breakthroughs of the Decade" collection [216]. In 2013, the exceptional significance of the optogenetic approach for neurobiological research was acknowledged by the prestigious Brain Prize awarded to six researchers who have made consequential contribution to the elaboration and development of optogenetic tools. Optogenetics has been included in the list of fundamental approaches to the implementation of the large research program BRAIN initiative (https:// braininitiative.nih.gov/) supervised by the National Institutes of Health. Furthermore, we believe that the success of optogenetics has had a global impact on the development of scientific methodology, as it has become a potent catalyst for the development of diverse genetically encoded tools. The several hundred breakthrough studies that have been published over the past decade and demonstrated the flexibility and efficiency of the new approaches (chemogenetics, thermogenetics, and hybrid photochemical methods) serve as the best evidence to support this assertion. In addition to these well-proven methods, fundamentally new approaches continue to appear, such as ultrasound neuronal stimulation mediated by cation activation (sonogenetics) [217] and single-component magnetic stimulation by iron-containing proteins (magnetogenetics) [218]. We wish these methods every success.

## Abbreviations

ADPR: adenosine diphosphate ribose
ATP: adenosine triphosphate
BL-OG: bioluminescent optogenetics
CHO: Chinese hamster ovary cells
CID: chemically induced dimerization
CNO: clozapine N-oxide
DAAO: D-amino acid oxidase
DHFR: dihydrofolate reductase
DREADDs: designer receptors exclusively activated by designer drugs
FAST: fluorescence-activating and absorption shifting tag
FLIRT: fast local infrared thermogenetics
FKBP: FK506 binding protein
FlyMAD: fly mind-altering device
FRB: FKBP12-rapamycin binding domain
GABA: gamma-aminobutyric acid
GFP: green fluorescent protein
GPCR: G-protein-coupled receptor
GR: gustatory receptors
hsp: heat shock protein
HEK293: human embryonic kidney 293 cells
IPD: ion pore domain
IR: infrared or ionotropic receptor (context-sensitive)
IR-LEGO: infrared-laser evoked gene operator
KOR: kappa-opioid receptor
LBD: ligand-binding domain
PhoCl: photo-cleavable
PSAM: pharmacologically selective actuator module
PSEM: pharmacologically selective effector molecule
PYP: photoactive yellow protein
RASSL: receptors activated solely by synthetic ligands
RNAT: RNA thermometer
TeNT: tetanus toxin
TRP: transient receptor potential
